# Image Parallel Encryption Technology Based on Sequence Generator and Chaotic Measurement Matrix

**DOI:** 10.3390/e22010076

**Published:** 2020-01-06

**Authors:** Jiayin Yu, Shiyu Guo, Xiaomeng Song, Yaqin Xie, Erfu Wang

**Affiliations:** Key Lab of Electronic and Communication Engineering, Heilongjiang University, Harbin 150080, China

**Keywords:** compressed sensing, initial sensitivity, parallel transmission, logic circuit

## Abstract

In this paper, a new image encryption transmission algorithm based on the parallel mode is proposed. This algorithm aims to improve information transmission efficiency and security based on existing hardware conditions. To improve efficiency, this paper adopts the method of parallel compressed sensing to realize image transmission. Compressed sensing can perform data sampling and compression at a rate much lower than the Nyquist sampling rate. To enhance security, this algorithm combines a sequence signal generator with chaotic cryptography. The initial sensitivity of chaos, used in a measurement matrix, makes it possible to improve the security of an encryption algorithm. The cryptographic characteristics of chaotic signals can be fully utilized by the flexible digital logic circuit. Simulation experiments and analyses show that the algorithm achieves the goal of improving transmission efficiency and has the capacity to resist illegal attacks.

## 1. Introduction

With the rapid development of data networks and information technology, people’s productivity and life are closely related to network technology, and the network has become the mainstream carrier of information [1]. There is a large amount of data transmitted in the network at any time, especially digital information, which is easy to store and forward, and noise is not cumulative, so the data can be widely transmitted, stored, and processed in the network [2]. The era of big data requires faster computing power and stronger storage capacity. Large amounts of data will bring about reliability and scalability problems, users may store huge amounts of historical data, and the data scale will continue to grow. Faced with such technical challenges, it is necessary to increase hardware speed, but it is also very important to turn algorithms more efficient. Simultaneously, security problems arising from data transmission are increasingly prominent. In the military and finance fields, there is a high demand for information security [3]. In this context, data encryption technology is increasingly researched by scholars. Digital images, with their unique characteristics compared to text information and voice information, are widely used in the fields of national defense, education, medical treatment, remote sensing, and environmental monitoring. Based on the above two problems, transmitting image information efficiently and safely in the vulnerable transmission network is particularly important [4].

There are many kinds of image encryption methods according to different standards. Such as static image encryption and adaptive image encryption algorithm; digital watermark image encryption and chaotic image encryption. Ref. [5] proposed a novel watermarking scheme addressed for medical image to ensure the security of functional magnetic resonance imaging (fMRI) data. In order to increase the hidden storage and enhance the robust of the image encryption algorithm. Yang et al. proposed a simple but robust digital watermarking for color image on Euclidean norms and quick coefficient alignment [6]. The above conventional encryption methods can guarantee security, in the process of encryption/decryption, but how to combine security with transmission efficiency has become the current problem.

In recent years, compressed sensing, as a cryptosystem, has attracted much attention owing to its low complexity and compressibility in the sampling process [7]. It is not only a data compression method but also a cryptographic system, which has attracted researchers’ attention. Compressed sensing can sample the compressible signal at the frequency far lower than that specified by Nyquist’s sampling theorem, and can ensure that the receiver can accurately reconstruct the original signal. It can effectively avoid resource waste, with a low sampling rate. Simultaneously, due to the randomness of the measurement matrix during the execution of compressed sensing, CS can provide a definite encryption function. Rachlin and Baron researched the security of the measurement matrix, and they proposed the computational notion of secrecy [8]. Orsemir and Altun proposed a cryptosystem based on the selection of a random measurement matrix and examined the robustness of the CS-based encryption algorithm and analyzed the algorithm based on compressed sensing is computationally secure [9]. Color image encryption algorithm was introduced by using CS with Arnold transform. The measurement matrix was scrambled by Anold [10]. Endra et al. compared the differences between random sensing matrixes and the optimized sensing matrix of the reconstructed process, and introduced a new CS algorithm [11]. Zhou et al. first compress the image and then encrypt the image, through a cyclic shift operation controlled by a hyper-chaotic system, to obtain a final plaintext image, and the cyclic shift operation is used to change the position of the pixels [12]. Currently, due to the low plaintext relevance of the above algorithm, the security of the algorithm is weak. George proposed an algorithm based on a linear feedback shift register and compressed sensing, which was validated through different block-based image. The algorithm has better performance [13]. Ref. [14] Presents a system about scrambling the CS audio data which combine with two-dimensional cellular automata.

To improve the computational efficiency of compressed sensing and the security of image encryption, a parallel image encryption technique based on a sequence signal generator was proposed. This algorithm is designed from the perspective of information security transmission and integrates new data to maximize the security of encryption technology. In order to reduce the encryption/decryption computation time and reducing the amount of information storage. A chaos system combines with a sequence signal generator is introduced for designing the CS measurement matrix. A chaotic system has initial value sensitivity and complex dynamic behavior, which can provide good randomness, correlation, and complexity of a pseudo-random sequence. However, when implemented on a finite precision computer, chaotic systems end up in dynamical degradation of chaotic properties. Ref. [15] Proposes a novel image encryption using finite precision error. The generated sequence has sufficient randomness to be used in image encryption. Ref. [16] Presents an encryption scheme based on pseudo-orbits of 1D chaotic maps. By combining chaotic cryptography with the classical cryptography concepts of scrambling and diffusion, chaotic systems have the characteristics of cryptography. This paper focuses on how to use the cryptographic characteristics of a chaotic system to optimize the process of CS. Additionally, the cryptographic characteristics of chaos are used to expand the key spacing and to enhance the efficiency and security of compressed sensing theory, to meet the requirements of image security and efficient transmission.

## 2. Theories and Methods

**Figure 1:**
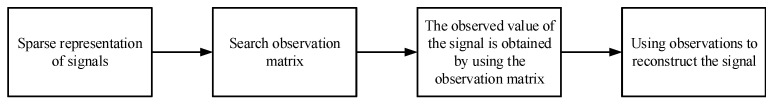
Block diagram of compressed sensing implementation.

The theory of compressed perception was formally proposed by E.J. Candes, in 2004 [17], and already had a definite theoretical foundation in the last century. This technology has been applied in many fields, including image processing, medical imaging, geophysics, computer science, signal processing, etc. CS is a technique that seeks sparse solutions of underdetermined linear systems. It breaks from the traditional Shannon sampling theorem and can obtain discrete samples under conditions including far less than the Nyquist sampling rate [18]. CS has two cores. One is signal sparsity, which means that a signal has a finite number of non-zero values, and the signal can be determined using those values. The second core is the uncorrelated feature, where the information is compressed by a non-adaptive sampling method [19], that the signal needs to be related to a set of determined waveforms, and the sparse space where the required signal is irrelevant to the waveforms. Figure 1 shows the CS implementation block diagram:

As can be seen from Figure 1, the compressed sensing process has three key steps. First, the original signal needs to be sparse. Secondly, the choice of measurement matrix is also very important. Different measurement matrices will produce different compression and encryption effects. Finally, the selection of reconstruction algorithm will directly affect the recovery of the original signal in the decryption process.

### 2.1. Mathematical Representation of Compressed Sensing

Suppose a two-dimensional signal *X* of size N×N is needed in the process of achieving compressed sensing to make the signal sparse. Under the corresponding sparse space of the signal, CS can achieve effective compression and sampling. Using Equation (1), CS can generate the sparse representation of the signal *X*, under [20]

(1) $$X=\sum_{n=1}^{N}\psi_{n}s_{n}=\psi s,$$

where ψ is the sparse basis matrix and *s* is the projection under the sparse basis ψ. In Equation (1), if there are K(K≤N) non-zero coefficients, the signal *X* is said to be compressible under a sparse basis ψ, and the sparsity is *K*. If there is a two-dimensional matrix ϕ of size M×N, then the original signal *X* can be converted into a signal of size M×N by the following Equation (2):

(2) Y=ϕX=ϕψS,

where *Y* is called the measurement value and ϕ is called the measurement matrix. On the basis of the known measurement value *Y* and measurement matrix phi, CS can obtain the original signal *X* by solving the underdetermined equation. In the traditional underdetermined equation, there should be infinite solutions; however, because *s* is sparse, conversion to an optimization problem is possible [21]. The unique optimal solution of the underdetermined equation can be found by obtaining the minimum norm in the following Equation (3):

(3) $${min∥s∥}_{0}\;s_{.}t_{.}\;Y=\phi \psi s,$$

where ∥m∥ represents $L_{0}$ norm, *s* is the recovery signal, and *Y* is the measurement signal; and, because *s* is obtained using a sparse basis transformation, the original signal *X* can be recovered from the signal *s* through a single inverse transformation.

In the process of CS, the following three actions are crucial:

1. Selection of a sparse basis: For a signal *X* of length *N*, select a sparse basis. If the *K* coefficient is not zero, after sparse transformation and K≤N, then we say the signal *X* is *K* sparse under the sparse basis [22]. The original signal can be recovered, using the sparse signal; however, the approximation of the original signal is obtained.
2. Design of a measurement matrix: in the design of a measurement matrix, the restricted isometry property (RIP) needs to be satisfied in order to solve Y=ϕX, which is an underdetermined solution problem. The RIP can guarantee the one-to-one mapping of original space to sparse space. In the algorithm proposed here, a chaotic signal is used to generate a measurement matrix, sequence signals generated by a sequence signal generator are then used to generate multiple measurement matrixes, and then, images are compressed and encrypted using multiple measurement matrixes.
3. Selection of a reconstruction algorithm: In the process of signal reconstruction, choosing an optimal reconstruction algorithm is key to the reconstruction effort. Currently, the reconstruction algorithm of CS is mainly divided into two categories: first, including a greedy algorithm, matching tracking algorithm, orthogonal matching tracking algorithm, etc. The second category includes a convex optimization algorithm, gradient projection method, basis tracking method, minimum Angle regression method, etc.

### 2.2. Logistic-Tent Chaotic System

In past work, Yu proposed to construct the measurement matrix of CS using chaotic mapping, and to generate chaotic sequences using logistic mapping and tent mapping [23]. Due to the ergodic characteristics of chaotic systems, the measurement matrix constructed by a chaotic system is essentially a sub-Gaussian random matrix. Additionally, the sub-Gaussian matrix can satisfy the RIP characteristics, thus it can be said that the chaos measurement matrix has good statistical characteristics. For a cryptography system based on the theory of CS, the chaotic system used to generate the measurement matrix should not only have good statistical characteristics, but also have good cryptography characteristics. Therefore, this paper adopts a compound chaotic logistic-tent system (LTS) to enhance the security intensity of the chaos matrix.

The LTS is generated by the combination of a logistic system and a tent system, both one-dimensional chaotic. By combining the logistic and tent subsystems expressed by Equations (4) and (5), the LTS is obtained. The combined system can generate chaotic sequences with chaotic characteristics. The combined system is expressed in Equation (6):

(4) $$Z_{n+1}=\mu Z_{n}\left(1-Z_{n}\right),$$

(5) $$Z_{n+1}=\left\{\begin{array}{ccc} \frac{Z_{n}}{p} & , & \;0<Z_{n}<p\\ \frac{1-Z_{n}}{1-p} & , & \;p\leq Z_{n}<1 \end{array}\right.$$

(6) $$Z_{n+1}=\left\{\begin{array}{ccc} {}[rZ_{n}\left(1-Z_{n}\right)+\frac{\left(4-r\right)Z_{n}}{2}]mod1 & ,\; & Z_{n}<0.5\\ {}[rZ_{n}\left(1-Z_{n}\right)+\frac{\left(4-r\right)\left(1-Z_{n}\right)}{2}]mod1 & ,\; & Z_{n}\geq 0.5 \end{array}\right.$$

where the control parameters of the logistic system mapping are μ∈[3.57,4], the control parameters of the tent system are ρ∈(0,1), and those of the composite system are r∈(0,4]. For the above three systems, all the initial values are $Z_{n}\in \left(0,1\right)$.

## 3. Parallel Compressed Sensing Encryption Algorithm Based on Sequence Generator

### 3.1. Algorithm Principle

In the process of image encryption and transmission, transmission is usually by row or column, with efficiency naturally depending on the dimension of the image. In order to improve the efficiency of encryption and transmission. This paper proposes a block partitioning, parallel compression sensing, and encryption transmission algorithm. Based on the sensitivity to initial values and the pseudo-randomness of the chaotic signals, the algorithm combines the cryptographic characteristics of chaos with the theory of the compressed sensing to generate the measurement matrix. Therefore, this algorithm can solve the problems of traditional compressed sensing’s low security, and the waste of large storage resources in reconstruction.

In order to improve the efficiency of information transmission and expand the key space. The algorithm will combine the theories of digital logic circuits and CS theory. The implementation of this algorithm is mainly divided into the following steps.

First, binary sequence signals of appropriate length will be generated using a signal sequence generator, and the binary sequence signals will be used as “modulation signals” to “slightly disturb” the initial value of the chaotic system. Owing to the sensitivity of the initial value of the chaotic system, any small change of the initial value will directly affect the entire chaotic matrix, such that the multiple chaotic matrices generated are guaranteed to be different from each other. In this paper, according to the modulation of the binary sequence, eight chaotic matrix will be needed to achieve the process, and the security of the image encryption is improved. Meanwhile, the digital logic circuit is flexible. By simply adjusting the structure of the logic circuit, different “modulation signals” can be generated to change the key.

Then, the chaos matrix, after “adding disturbance”, is taken as the measurement matrix, and compressed sensing is adopted for the image encryption. Here, the 256×256 image is divided into eight blocks according to the columns, and the image is segmented and compressed in parallel. Appropriate partitioning can improve the security and transmission rate of the algorithm.

**Figure 2:**
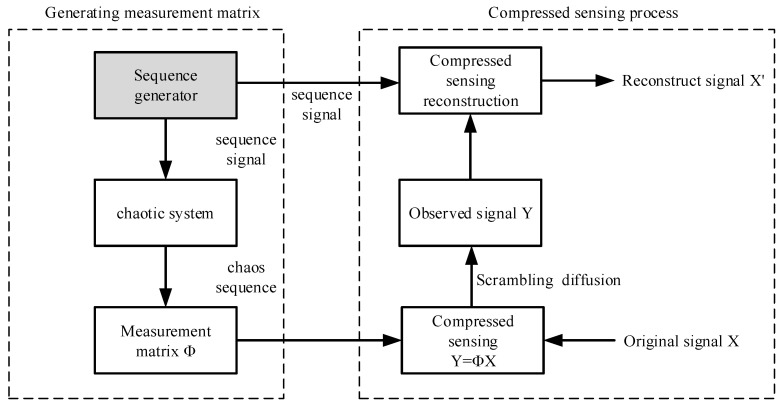
Parallel compression sensing encryption algorithm based on sequence generator.

Finally, in order to show better cryptographic characteristics, the sampled cipher text images are confused and scrambled. Thus, the energy blocks measured by the same measurement matrix can be evenly distributed over the whole image, to achieve the efficient transmission and effective encryption of the image information. The block diagram for this algorithm is given as Figure 2:

The left side of Figure 2 describes the measurement matrix generation process. Firstly, the logic circuit generates the sequence signal, and the chaotic matrix is generated by the chaotic system under the modulation of the sequence signal. The sequence signal is transmitted to the decryption end as the key. The right part describes the process of measurement and reconstruction. Since the sparse image is divided into eight blocks above, eight measurements are required in this part. The results of multiple measurements are combined to form an observation signal. The signal is reconstructed using an orthogonal pursuit (OMP) algorithm. The sequence signal generator and the parallel compressed sensing scheme in Figure 2 are described in detail below.

### 3.2. Sequence Signal Generator Mode

**Figure 3:**
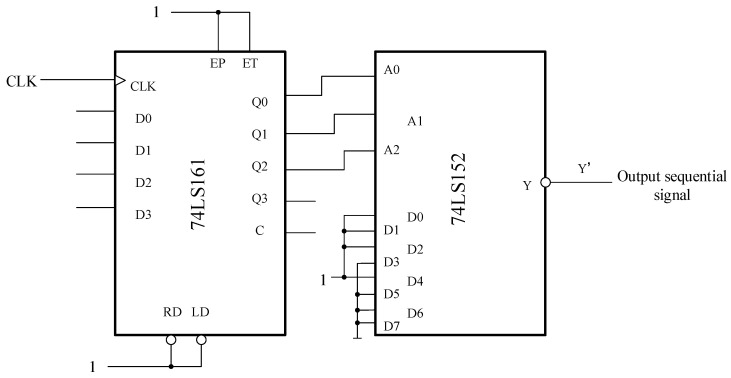
Circuit diagram of sequence signal generator.

Here, the sequence signal generator shown in Figure 3 is designed and consists of the synchronous hexadecimal addition counter 74LS161 and the 8-to-1 data selector 74LS152. For example, if one needs to generate an eight-bit sequence signal 00010111 (the time sequence is from left to right), can use an octal counter that can be used along with an 8-to-1 data selector. The octal counter is taken from the lower three bits of counter 74ls161, a four-bit binary counter. Connecting the two parts provides the circuit of the sequence signal generator, as shown in Figure 3:

Clock signals are continuously added to the counter, and the state of $Q_{2}Q_{1}Q_{0}$ is continuously cyclic according to the order in Table 1. The eight binary digits (1 or 0) of data selectors $D_{0}$–$D_{7}$ can be used as keys to modulate the initial value of the chaotic system. It should be noted that only the high and low bits of $D_{0}$–$D_{7}$ need to be modified to generate different sequence signals, thus the circuit has flexible and convenient characteristics. The sequence signal generation in this paper is shown in Table 1.

Here, the initial value of the chaotic LTS is chosen to be 0.32568749. When the output of the binary sequence signal generator is 1, the small perturbation $10^{-8}$ of the initial value ensures that it is still in a chaotic state within the image block.

### 3.3. Parallel Compression Sensing

**Figure 4:**
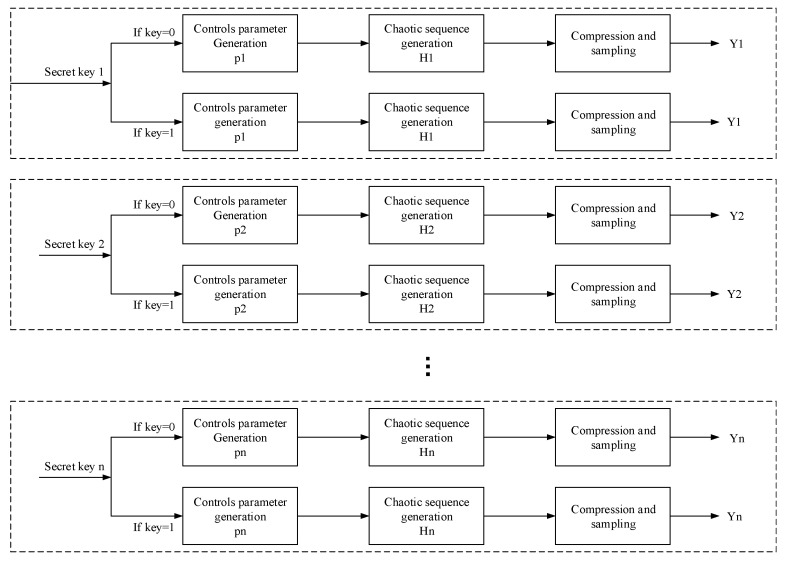
Parallel sampling compression of compressed sensing process.

As above, the binary sequence generated by the sequence signal generator is used to fine-tune the initial value of the chaotic system, and the generated chaotic matrix is then used as the measurement matrix of the CS. A specific compression ratio is set to generate a measurement matrix, and the sparse image is compressed and sampled. Here, the sparse plaintext image is evenly cut into eight blocks. The size of each block is 256×32, and the parallel transmission of the eight blocks is greatly improved in efficiency, compared with the transmission, by 256 columns. The process of CS using the generated measurement matrix is shown in Figure 4:

Where $P_{1},P_{2},\ldots ,P_{n}$ is the control parameter of chaotic system. Under the influence of the control parameter, the chaotic matrix is generated as the measurement matrix $H_{1},H_{2},\ldots ,H_{n}$. In this paper, eight measurement matrices will be generated for the corresponding eight plaintext images, and parallel measurements will be made for each block to obtain the measurement value $Y_{1},Y_{2},\ldots ,Y_{n}$.

Converse to other chaotic compressed sensing systems, this algorithm does not need to transmit the measurement matrix from the sending end in the reconstruction process. After using the measurement matrix at the sending end, the algorithm does not need to store it. It only needs to use the same sequence signal generator at the receiving end to fine-tune the initial value of the chaotic system, so that the measurement matrix can be reproduced at the receiving end. Then, the reconstruction process of the cipher text image can be completed. Therefore, this algorithm can greatly save storage resources and effectively avoid resource waste.

It should be noted that the parallel-based compressed sensing image encryption scheme can effectively and reliably complete image encryption, but it also has drawbacks. Since the block of the plaintext image is sampled, the energy of each block in the measured value is stored intensively. To overcome this defect, we use a diffusion and scrambling operation to force the energy of cipher text image to be evenly distributed over the whole image.

## 4. Simulation Results and Security Analysis

We selected the gray image “pepper,” of size 256×256, from the standard test library. First, discrete wavelet transform (DWT) was used to sparse the image, and then the image was divided into 8 parts of size 256×32. Then, a sequence signal generator was designed to generate sequence signal 00010111. According to this signal, the initial chaos value is fine-tuned, with step size $10^{-8}$, and the chaos matrix is taken as the measurement matrix. CS is used to compress and encrypt the 8 sub-blocks in parallel. The dimension of the measurement matrix in the encryption process is 190×256, i.e., the compression ratio is 74.2%. Finally, the encrypted cipher text image is diffused, and the reference Formula (7) is given as follows:

(7) $$Q^{*}\left(n\right)=Q\left(N\right)⊕k_{d}\left(n\right)⊕Q^{*}\left(n-1\right)$$

**Figure 5:**
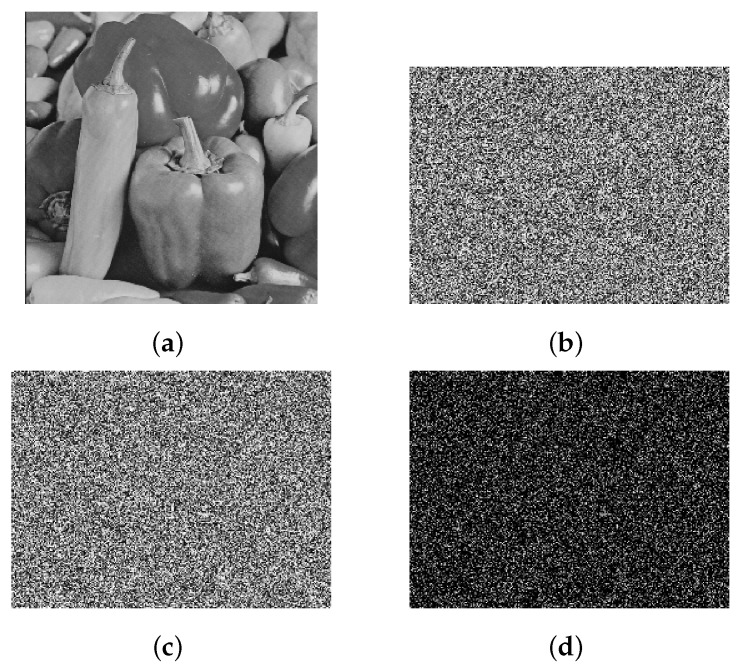
Results of gray image parallel compression perception encryption. (**a**) Original image, (**b**) compressed sensing encrypted image, (**c**) diffused cipher text image, (**d**) difference between (**b**) and (**c**).

The simulation results for the above-proposed algorithm are shown in Figure 5:

From the simulation results above, it can be seen that the encrypted image in this paper is like a snowflake, and no valid information about plaintext can be distinguished visually by observation. From the perspective of subjective judgment, it can be seen that the algorithm achieves the compression and encryption of the plaintext image. Next, we will describe the reconstruction and restoration of the image; compare the algorithm proposed here with other compression sensing encryption algorithms; and, provide the algorithm’s ability to resist exhaustive attack, anti-differential attack, tailoring attack, and noise attack.

### 4.1. Encryption Performance Analysis

**Figure 6:**
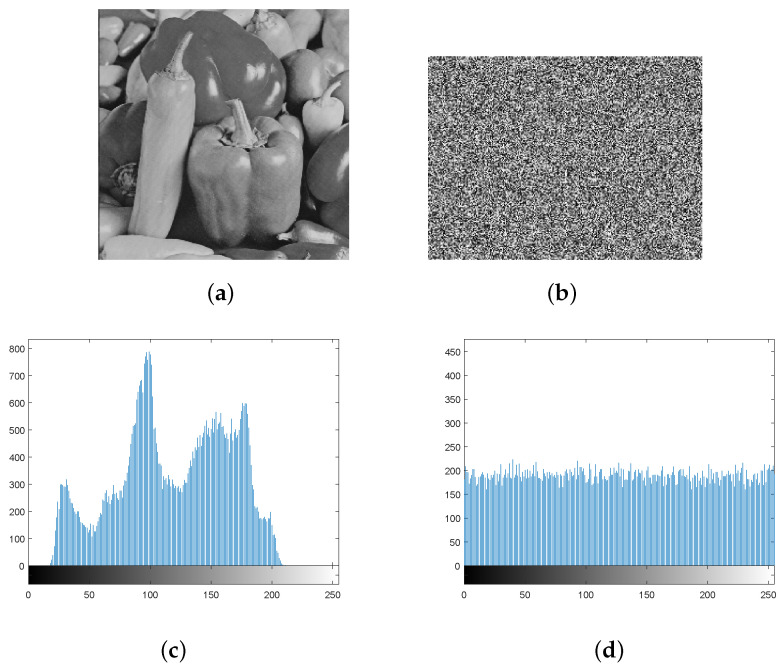
Histogram of encryption and decryption image. (**a**) Original image, (**b**) cipher image, (**c**) histogram of plaintext image, (**d**) Histogram of cipher text image.

A histogram can directly reflect the distribution of the pixel intensity values in the image. The histograms of the cipher images should possess a similar distribution when the encryption algorithm is effective. Figure 6a,b present plaintext images and cipher text images, respectively, while Figure 6c,d are the histograms of the two respective images.

It can be seen that the pixel value of the cipher text image is evenly distributed in the interval [0,255], which is completely different from the normal image, indicating that the attacker cannot obtain any valid information about the original image from the histogram of the encrypted image.

Information entropy can measure the distribution of the gray value in the image. The more uniform the gray distribution is, the larger the information entropy of the image will be. When the information entropy of the image is low, it is easy for the image to be maliciously attacked and tampered with by criminals. Equation (8) which calculates information entropy is as follows:

(8) $$H\left(m\right)=-\sum_{i=1}^{n}p\left(m_{i}\right)\log_{2}p(m_{i}),$$

where *m* is the pixel collection. $p(m_{i})$ is probability of occurrence of *m* and *n* is a total number of $m_{i}$. For encrypted images, the higher the information entropy, the more uniform the energy distribution in the image, and the less useful information an attacker can get from the gray distribution. The entropy of this algorithm is listed as Table 2:

Table 2 shows the change of the image entropy, when the compression ratio changes, in the process of compression and encryption. The image entropy value after encryption in this paper is close to 8, which can achieve effective encryption.

The correlation of adjacent pixels in the original image can reflect the diffusion degree of pixels in the image, and the correlation of adjacent pixels in the encrypted image should be close to 0. The correlation coefficients between adjacent pixels *x* and *y* is defined as Formula (9):

(9) $$\rho_{xy}=\frac{cov(x,y)}{\sqrt{D(x)D(y)}}$$

where cov(x,y)=E[x−E(x)][y−E(y)]; E(x) and E(y) are the average values of *x* and *y*. D(x) and D(y) are the standard deviations of *x* and *y*.

In the literature [12], the image is compressed from two directions using the fractional-order Merlin transform method, while as in [24], the image is encrypted from orthogonal directions using the discrete fractional-order random measurement matrix. In this paper, the correlation of adjacent pixels is compared with the above two literatures. It can be seen from Table 3 that this algorithm has lower similarity and is better than the image encryption process in the literatures.

**Figure 7:**
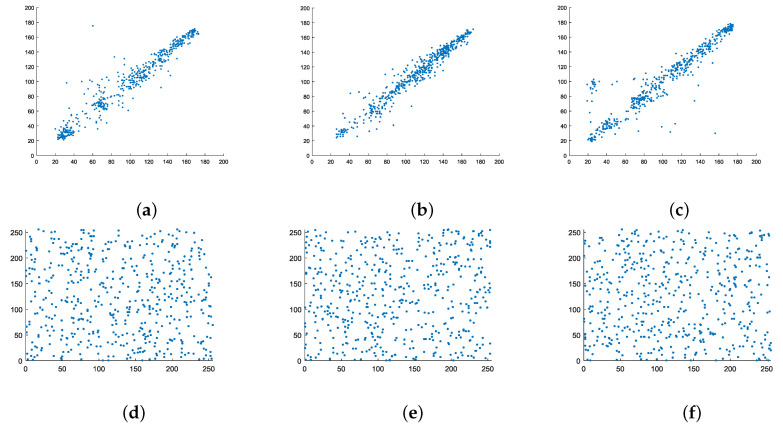
Distribution of adjacent pixels. (**a**) Plaintext horizontal adjacent pixels, (**b**) plaintext vertical adjacent pixels, (**c**) plaintext diagonal adjacent pixels, (**d**) cipher text horizontal adjacent pixels, (**e**) cipher text vertical adjacent pixels, (**f**) cipher text diagonal adjacent.

Figure 7 shows the correlation distribution between the adjacent pixels of the original image and the adjacent pixels of the encrypted image. It can be seen from Figure 7 that in the original image, the correlation between the adjacent pixels is very high, while the correlation between the adjacent pixels of the encrypted image is very low. According to the results, using data and images, the algorithm proposed here achieves good encryption in terms of the correlation degree of adjacent pixels.

### 4.2. Decryption (Reconstruction) Performance Analysis

Image decryption can be regarded as the inverse operation of image encryption. Here, CS is used to achieve encryption, and the decryption process is also called the reconstruction process. First, an anti-diffusion operation should be carried out on the cipher text image. The expression is shown in Equation (10). The receiver uses the reconstructed measurement matrix to generate the sequence signal, and the initial value control parameters of the chaos matrix, according to the key held, to restore the chaos matrix and acquire the measurement matrix needed in the reconstruction process. Finally, the sparse signal is reconstructed by the RIP optimization criterion given in Equation (11), and the plaintext signal is recovered using Equation (12):

(10) $$Q\left(n\right)=Q^{*}\left(n\right)⊕Q^{*}\left(n-1\right)⊕k_{d}\left(n\right)$$

(11) $${\hat{s}}_{i}=\arg\min_{s_{i}\in R^{N}}{∥s_{i}∥}_{1}\;s_{.}t_{.}\;{\hat{y}}_{i}=\phi_{i}x_{i}=\phi_{i}\psi_{i}s_{i}\;i=1,\ldots N$$

(12) $${\hat{x}}_{i}=\psi {\hat{s}}_{i}.$$

**Figure 8:**
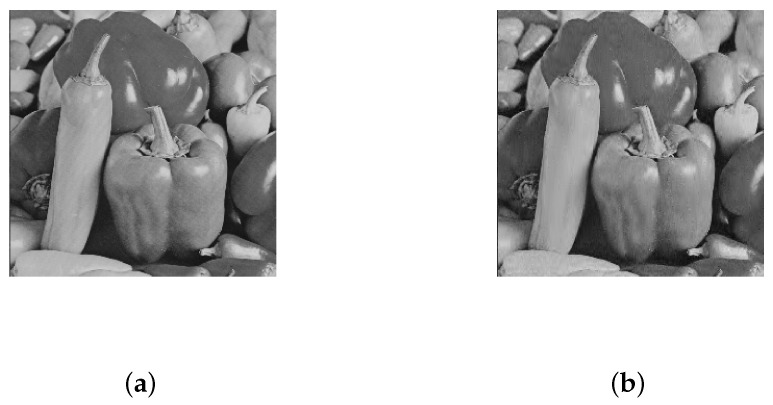
Encrypted image reconstruction. (**a**) Original image, (**b**) reconstructed image.

Accordingly, the reconstruction results are shown in Figure 8:

As can be seen from Figure 8, the reconstructed image can show the effective information of the original image. Subjectively, we can allow that the algorithm in this paper achieves image restoration. Natural images have very high structural similarity, which is reflected in the strong correlation between the pixels of images, and structural similarity is an index to measure the similarity of two images. The value range of structural similarity is 0 to 1. When the similarity is close to 1, the two images are more similar; otherwise, the two pictures are quite different. It is calculated as follows:

(13) $$SSIM=\frac{\left(2\mu_{X}\mu_{Y}+C_{1}\right)\left(2\sigma_{XY}+C_{2}\right)}{\left(\mu_{X}^{2}+\mu_{Y}^{2}\right)\left(\sigma_{X}^{2}+\sigma_{Y}^{2}+C_{2}\right)},$$

where $C_{1}={\left(k_{1}\times L\right)}^{2}$, $C_{2}={\left(k_{2}\times L\right)}^{2}$, $k_{1}=0.03$, $k_{2}=0.03$, L = 255, and $\mu_{X}$, $\mu_{Y}$, $\sigma_{X}$, $\sigma_{Y}$, $\sigma_{XY}$ represent the mean, variances and covariance of the plain image and cipher image. Table 4 shows the degree of structural similarity between the original image and the reconstructed image, under different compression rates.

As can be seen from Table 4, as the compression ratio of the plaintext image increases, the image similarity also increases, indicating that the image has been effectively restored. However, the similarity of cipher text images is very low, which proves that the cipher text images meet the requirements of image encryption.

**Figure 9:**
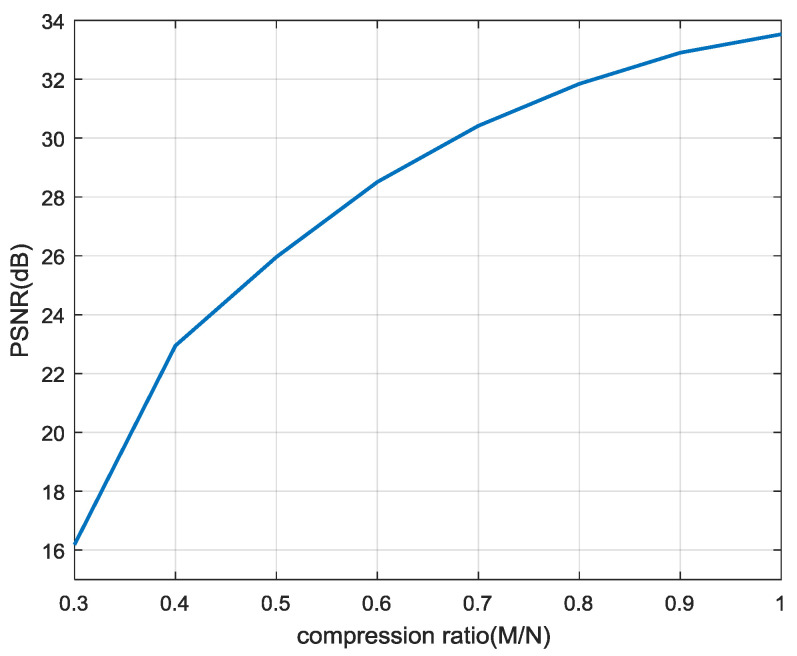
PSNR of reconstructed image.

The peak signal-to-noise ratio (PSNR) represents the ratio between the maximum possible power of a signal and the destructive noise power that affects its signal accuracy. It can be defined by the mean squared error (MSE), and its equation is as Formula (14):

(14) $$PSNR=10\log_{10}\frac{L^{2}}{MSE},$$

where *L* is the value range of the gray scale in the image. For an eight-bit image the value range is L=256. Generally, the higher the PSNR of the image, the lower is its distortion degree. Figure 9 shows the PSNR of the image, reconstructed by the algorithm in this work for different compression rates.

As can be seen from Figure 9, the value of PSNR increases with the increase of the compression rate. Moreover, the PSNR of the images reconstructed by this algorithm can meet the requirements of image recovery. When the compression ratio is greater than 50%, the algorithm can achieve a better recovery effect. Therefore, the compression rate can be set reasonably according to different requirements.

### 4.3. Key Sensitivity Analysis

**Figure 10:**
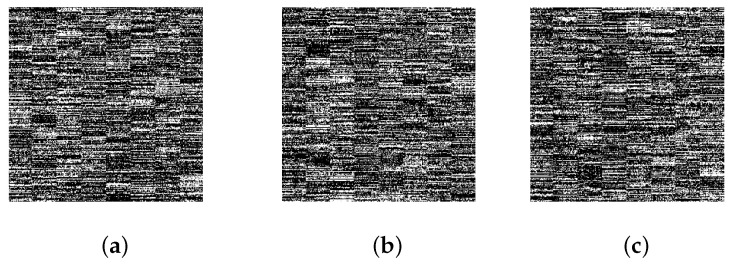
Key sensitivity analysis. (**a**) Initial value change $10^{-14}$, (**b**) initial value change $10^{-15}$, (**c**) initial value change $10^{-16}$.

Key sensitivity refers to the degree to which the cipher text changes when the initial key changes slightly. The sensitivity of the initial value of the chaos can be used to detect the sensitivity of the algorithm. When the initial value of the chaotic system is small, changing the key means the reconstructed image at the receiving end will be greatly different from the original. Figure 10a is the reconstructed image when the order of magnitude of the key changes by $10^{-14}$, Figure 10b is the recovered image when the order of magnitude changes by $10^{-15}$, and Figure 10c is the image when the order of magnitude changes by $10^{-16}$.

It can be seen that, although the initial value in the reconstruction process only changes very slightly, we can no longer observe any effective information from the original image in the reconstructed image, which proves that this algorithm has good key sensitivity.

### 4.4. Safety Analysis

In image encryption, the key spacing should be large enough to resist various violent attacks. The above key sensitivity experiment also shows that the encryption algorithm needs to have a strong dependence on the key. If the decryption key changes slightly, the decrypted image will be greatly different from the original image. As an important reference for evaluating an encryption algorithm, the key spacing directly determines the algorithm’s ability to resist an exhaustive attack.

For the algorithm proposed here, without considering the placement and diffusion process, and only considering the impact of the measurement matrix on decryption. There are a total of two encryption algorithm keys. One is the sequence generator with a total of eight parameters. So the key 1 is {$Y_{0}^{\prime },Y_{1}^{\prime },Y_{2}^{\prime },Y_{3}^{\prime },Y_{4}^{\prime },Y_{5}^{\prime },Y_{6}^{\prime },Y_{7}^{\prime }$}. The other is the control parameters of the chaos is the key 2 {μ}. Since there are nine parameters in the key space in this paper. According to the international standard IEEE 754, the index portion is expressed as a positive value to simplify the comparison. The significant digit of a double-precision floating-point type is 52 bits. The key space is greater than $2^{52\times 9}=2^{468}$, and it can be seen from the key space analysis that this key space is highly resistant to exhaustive attack and has high encryption security.

The sensitivity of the encryption algorithm to the plaintext can determine the ability of the algorithm to resist differential attacks. The parameters used to measure this sensitivity can be the number of pixels change rate (NPCR), for normalized pixels, or the description of unified average changing intensity (UACI). The equations for NPCR and UACI are given as Equations (15) and (16):

(15) $$NPCR=\frac{1}{N\times M}\sum_{i=1}^{M}\sum_{j=1}^{N}E\left(i,j\right)\times 100\%$$

(16) $$UACI=\frac{1}{N\times M}\sum_{i=1}^{M}\sum_{j=1}^{N}\frac{|M_{1}\left(i,j\right)-M_{2}\left(i,j\right)|}{255}\times 100\%,$$

where *M* and *N* are the number of rows and columns of image pixels. The NPCR and UACI values of the proposed algorithm are calculated in Table 5.

In literature [25], the chaotic generation key is used as the index of the row and column replacements in the image encryption process, and the encryption method of row and column replacement is adopted to encrypt the image. The work in literature [26] adopts a hyper chaotic system based on a closed-loop modulation to replace image pixels. In literature [27], piecewise linear chaotic mapping is adopted to exchange binary elements in the original image sequence, using a chaotic sequence to scramble and encrypt the image. The NPCR and UACI evaluation criterion given in literature [28]. As seen in Table 5 and the NPCR and UACI values of the image encrypted by the proposed algorithm were close to the critical values: $N_{0.05}^{*}=99.5693\%$, $N_{0.01}^{*}=99.5527\%$, $N_{0.001}^{*}=99.5341\%$; where the critical values of UACI are $U_{0.05}^{*+}=33.6447\%$, $U_{0.05}^{*-}=33.2824\%$, $U_{0.01}^{*+}=33.7016\%$, $U_{0.01}^{*-}=33.2255\%$.

Therefore, compared with other algorithms, the algorithm in this work can resist differential attacks more effectively.

## 5. Conclusions

In this paper, an image parallel encryption technology based on sequence generator and chaotic measurement matrix is proposed, combines the stochastic characteristics of chaotic signals with a compressed sensing algorithm, which greatly expands the key spacing. By combining a digital logic circuit, this algorithm has more flexibility and security. At the same time, it also provides a new idea for the implementation of traditional encryption methods on hardware. The feasibility of the algorithm is verified by simulation, and the experimental results are analyzed comprehensively. This algorithm has a very high key sensitivity and can encrypt the image information very well. At the same time, the security analysis verifies that this algorithm can resist a brute force attack, such as an exhaustive attack or a differential attack.

In order to calculate the time required for the implementation of this algorithm. The experiment was repeated for 20 times, then average the results of the above experiments to avoid outliers. The algorithm encryption process takes 0.53 s, and the use of common compression perception algorithm encryption requires approximately 1 s. The algorithm decryption process takes 8.2 s, and the ordinary compression perception algorithm decryption requires 10 s; thus, the proposed algorithm uses the method of parallel transmission to effectively improve the efficiency of information transmission. Furthermore, the influence of noise on the algorithm is not considered in this paper. If there is noise interference, with different distribution characteristics, it remains to be studied whether the compressive sensing has a certain anti-noise capability, or whether the compressive sensing framework can improve the anti-noise capability through an optimization algorithm.

## Figures and Tables

**Table 1:** Circuit state conversion table.

CLK	$Q_{2}$	$Q_{1}$	$Q_{0}$	$Y^{\prime }$
0	0	0	0	${D_{0}}^{\prime }\left(0\right)$
1	0	0	1	${D_{1}}^{\prime }\left(0\right)$
2	0	1	0	${D_{2}}^{\prime }\left(0\right)$
3	0	1	1	${D_{3}}^{\prime }\left(1\right)$
4	1	0	0	${D_{4}}^{\prime }\left(0\right)$
5	1	0	1	${D_{5}}^{\prime }\left(1\right)$
6	1	1	0	${D_{6}}^{\prime }\left(1\right)$
7	1	1	1	${D_{7}}^{\prime }\left(1\right)$
8	0	0	0	${D_{0}}^{\prime }\left(0\right)$

**Table 2:** Information entropy of encrypted images.

Entropy	Compression Ratio							
	0.3	0.4	0.5	0.6	0.7	0.8	0.9	1
Cipher image	7.9903	7.9934	7.9939	7.9957	7.9958	7.9963	7.9974	7.9968

**Table 3:** Correlation between adjacent pixels of cipher text image.

Algorithm	Horizontal Direction	Vertical Direction	Diagonal Direction
Proposed algorithm	−0.0065	0.0073	0.0042
Ref. [12]	0.0586	−0.0021	0.0269
Ref. [24]	0.0597	−0.0766	0.0083

**Table 4:** Information entropy of encrypted images.

SSIM	Compression Ratio							
	0.3	0.4	0.5	0.6	0.7	0.8	0.9	1
Reconstructed image	0.4302	0.6129	0.7349	0.8388	0.9033	0.9424	0.9645	0.9801
Cipher image	0.0028	0.0032	0.0045	0.0047	0.0065	0.0071	0.0076	0.0085

**Table 5:** Correlation between adjacent pixels of cipher text image.

Index	Our Scheme	Ref. [25]	Ref. [26]	Ref. [27]
NPCR (100%)	99.6094	99.6075	99.6063	99.6198
UACI (100%)	33.4635	33.4195	33.3437	32.8014

## Abbreviations

The following abbreviations are used in this manuscript:

MDPI	Multidisciplinary Digital Publishing Institute
DOAJ	Directory of open access journals
TLA	Three letter acronym
LD	linear dichroism
